# Neuropsychiatric disorders in Parkinson’s disease

**DOI:** 10.1177/17562864251356062

**Published:** 2025-07-28

**Authors:** Beatrice Heim, Atbin Djamshidian

**Affiliations:** Department of Neurology, Medical University of Innsbruck, Innsbruck, Austria; Department of Neurology, Medical University of Innsbruck, Anichstrasse 35, Innsbruck 6020, Austria

**Keywords:** impulse control disorder, neuropsychiatric symptoms, Parkinson’s disease

## Abstract

Neuropsychiatric symptoms, such as depression, anxiety, cognitive changes, apathy or hallucinations are common in patients with Parkinson’s disease (PD). They can appear at any stage of the disease and some symptoms may even be a harbinger of PD. These neuropsychiatric complications often become more pronounced as PD progresses and may worsen particularly during ‘off-periods’. Moreover, neuropsychiatric symptoms are also frequently seen in patients with addictive behaviours, collectively called impulse control disorders, where insight is particularly low. In this narrative review, non-pharmacological as well as experimental and pragmatic pharmacological approaches are outlined to provide a deeper understanding of the best treatment strategy for these patients. Early detection as well as a tailored multidisciplinary approach is necessary to improve symptoms and ultimately the quality of life for patients and their family members.

## Introduction

Parkinson’s disease (PD) is primarily characterised by motor symptoms such as bradykinesia combined with either rest tremor, rigidity or both.^1^ However, neuropsychiatric symptoms are increasingly recognised as significant contributors to the overall morbidity. As such PD is now being sometimes also regarded as a complex neuropsychiatric disorder.^2^ The range of these neuropsychiatric symptoms is broad and includes disorders of affect (such as depression and anxiety), perception and thought processes (i.e. psychosis and dementia) and motivation (i.e. apathy and impulse control disorders (ICDs)). These symptoms can become more debilitating as the disease progresses, and may be more prominent during off-periods.

While neuropsychiatric symptoms can present in isolation, particularly in the early disease stage, they frequently co-occur and overlap with disease progression (e.g. depression in addition with anxiety or apathy).^2^ Typically, PD progresses through distinct phases. In the prodromal phase, various neuropsychiatric symptoms including depression, anxiety, REM sleep behaviour disorder (RBD), hyposmia and autonomic dysfunction – may occur and can precede the classical motor symptoms by several years. The emergence of motor symptoms, notably bradykinesia accompanied by rest tremor and/or rigidity, marks the beginning of the clinical motor phase of PD. These motor symptoms progress gradually and may eventually reach advanced stages characterised by motor fluctuations and treatment-resistant axial symptoms such as freezing of gait, postural instability and falls. Non-motor symptoms, as well as non-motor fluctuations are also frequent in advanced PD and significantly impair quality of life of patients and their family members.^3,4^ Therefore, it is not surprising that non-motor symptoms are key drivers for nursing home admission and increased morbidity as well as costly for society.^5^ Thus, early detection, an individual management and clinician’s vigilance to screen for these debilitating comorbidities is of paramount importance.

### General management

Tackling neuropsychiatric symptoms in PD is often challenging and a multidisciplinary team including psychologists, psychiatrists, neurologists, occupational therapist and physiotherapists may be necessary. Effective management should also include careful attention to comorbidities such as chronic pain or autonomic problems (e.g. bladder or bowel dysfunction) and insomnia. Clinicians are encouraged to proactively enquire patients, caregivers and family members about the presence of neuropsychiatric symptoms at every clinical visit to ensure early recognition and timely management.

### Optimisation of dopaminergic therapy

Individualised optimisation of drug therapy is recommended for all patients with PD. This is particularly important in those experiencing motor complications. Adjustments of dopaminergic therapy can also significantly alleviate several non-motor symptoms associated with ‘off-periods’ including anxiety and depression. In some PD patients, advanced therapies, such as continuous delivery of levodopa or apomorphine, may also effectively reduce non-motor symptoms as well as non-motor fluctuations.^6,7^ Conversely, peak-dose non-motor complications including psychosis, agitation and euphoria often improve with the overall reduction of dopaminergic medication.^8,9^ In line with this, a recent meta-analysis also showed that optimising motor performance may improve at least some neuropsychiatric symptoms. For example, scores for anxiety as well as depression were lower in those who underwent deep brain stimulation (DBS) of the sub-thalamic nucleus (STN).^10^ However, careful screening prior to STN-DBS should be performed and those at risk for suicide should be excluded.^11^

### Recognition of non-motor fluctuations

Non-motor fluctuations are often underdiagnosed and can be categorised into neuropsychiatric, sensory and autonomic subtypes.^12^ Particularly mood swings with anxiety, depression, feeling of panic,^13^ apathy, euphoria, disinhibition or confusion may be present in up to two-thirds of PD patients with motor fluctuations. Moreover, autonomic fluctuations with urinary urgency, sweating and sensory disturbances with pain are also frequently seen. Non-motor fluctuations are more common in female patients and increase with disease duration marking the onset of advanced PD.^12^ Typically, non-motor fluctuations develop simultaneously with or later than motor-fluctuations. Only in a minority of patients (<2%) non-motor-fluctuations may precede motor fluctuations.^14^ These non-motor fluctuations may have an even greater negative impact on the patient’s independence and quality of life than motor fluctuations. Early identification of non-motor fluctuations is crucial and highlights the urgent need for systematic, large-sample studies to develop better tools for detecting and treating these debilitating symptoms.^15^

### Depression and anxiety

The most frequently encountered mood disorders in PD are depression and anxiety.^5,16,17^

Apart from increased caregiver burden, depression in PD is also associated with disease progression, disability and cognitive decline.^5,18,19^ Surprisingly, no such association was found with anxiety. This is particularly interesting, as both anxiety and depression frequently co-occur.^19^

The prevalence rates of depression and anxiety varies from study to study probably due to the differences in scales used. However, it is thought that depression occurs in at least 30% and up to 50% of PD patients,^20,21^ with major depression being found in about 20%.^22^

Similarly, the prevalence rate of anxiety varies between 20% and almost 50%^17^ (Table 1). Both conditions are commonly underdiagnosed, partly due to symptom overlap with PD itself (e.g. fatigue, weight changes).^22^ Both can occur at any stage of the disease including the prodromal phase.^19^

**Table 1.** Prevalence and risk factors of neuropsychiatric symptoms and insomnia in Parkinson’s disease.

Symptom	Prevalence	Risk factors	References
Depression	30%–50%	Female gender, previous depression, severe motor symptoms	20, 21
Anxiety	20%–50%	Younger age, financial strain, female gender	17
Apathy	~40%	Advanced disease stage, cognitive impairment, post-DBS	66
ICDs	~15%–30% (with dopamine agonists)	Dopamine agonists, younger age, male gender, novelty seeking	8
Visual hallucinations	up to 75% (over disease course)	Cognitive impairment, longer disease duration, dopaminergic therapy	143
Psychosis	~20%	Advanced age, high-dose dopaminergic medication, dementia	163
Insomnia	60%–90%	Depression, anxiety, ICDs, dopaminergic fluctuations	102, 111, 112
RBD	33%–50%	Male gender, older age, cognitive impairment	164
MCI	10%–20% at diagnosis	Older age, severe motor symptoms, early psychotic symptoms	119
Dementia	Up to 80% after 20 years	Older age, longer disease duration, early psychotic symptoms, *GBA*/*APOE* mutations	123

DBS, deep brain stimulation; ICDs, impulse control disorders; MCI, mild cognitive impairment; RBD, REM sleep behaviour disorder.

While depression is more commonly reported in female patients, no such association was found for anxiety. Generally, in PD anxiety appears more prevalent in younger patients, possibly due to external factors such as financial strains or familial responsibilities.^19,23^

Recent evidence indicates that anxiety is intrinsic to PD^24^ and may serve as a risk factor for PD development, with retrospective data showing at least a doubled risk in anxious patients aged 50 and above.^25^ In line with this, anxiety is linked with PD severity, but not with PD duration, and is more common in those with falls and gait dysfunction than those with tremor-dominant PD.^26^ Anxiety scores are also typically higher during ‘off periods’.

Anxiety^27,28^, and in some studies also depression, may also be risk factors for cognitive decline.^29^ In addition, depression in the early stages of PD has been linked to worse motor prognosis.^30^ Typically, however, depression is persistent and worsens with disease progression. In line with this, depression and anxiety are particularly common in patients with motor fluctuations, those with prolonged off periods as part of non-motor fluctuations and those with dyskinesias.^2,22,26,31^ Symptoms intrinsic to PD, such as paucity of facial movements due to hypomimia or a stooped posture can mimic mood disorders and may lead to misdiagnoses (‘pseudo-mood disorders’). Clinicians must maintain awareness of these distinctions to avoid unnecessary treatment and ensure accurate diagnosis and targeted management.^32^ Moreover, depression and anxiety may arise as patients have been diagnosed with a chronic illness and fear of disease progression may be one underlying trigger factor.

### Pathophysiology

While the underlying aetiology is complex and the mechanisms are still incompletely understood, several factors including genetics and changes of the neurotransmitters due to neurodegeneration, medication status as well as psychosocial issues are thought to contribute to the development of these two mood disorders.^33^ For example a first degree family history of depression, previous history of anxiety and/or depression, female sex, REM sleep behaviour abnormalities as well as cognitive impairment and worse activity of daily living have been identified as risk factors.^22,34^ Moreover, depression is particularly more common in PD patients with the *LRKK2* G2019S mutation^31^ and in those with *GBA* mutations.^35^ The reason remains unknown, however, some studies also showed other neuropsychiatric complications such as hallucinations in genetic PD, suggesting a more limbic involvement.^31^

Apart from dopaminergic deficits dysfunction of noradrenaline and serotonergic pathways are thought to contribute to the development of depression.^33^ In fact, degeneration of serotonergic raphe neurons in the median and dorsal raphe nucleus can be more severe and occur prior to the dopaminergic cell loss in the substantia nigra.^36^ In line with this, histopathological studies showed degeneration of the locus coeruleus and of the raphe nucleus, the source of noradrenaline and serotine, respectively.^37^

Moreover, a widespread striatal and limbic dysfunction with white matter loss in the anterior cingulate as well as lower grey matter density in the orbitofrontal cortex, the rectal gyrus, the medial temporal, the anterior and medial cingular gyrus, as well as the parahippocampal gyrus was found in depressed PD patients.^38,39^ Similarly, in PD patients with anxiety, changes in the fear circuit and the cortico-striato-thalamocortical limbic circuit was found. More specifically, severity of anxiety is correlated with a reduction of grey matter volume of the amygdala and the anterior cingulate cortex as well as an increased functional connectivity between the amygdala and orbitofrontal cortex and hippocampus. Moreover, a reduced dopaminergic and noradrenergic activity in striatum, thalamus and locus coeruleus and a reduced serotoninergic activity in the thalamus was found.^40^

Taken together these imaging data suggest that depression and anxiety may arise from structural changes within the limbic cortico-striato-thalamocortical circuits.

Another hypothesis suggests that inflammatory activation by gut microbiota may play a role in developing PD but also neuropsychiatric symptoms such as depression and anxiety.^41^ More specifically alteration in gut microbiota composition can influence cytokines such as interleukin 6 (IL-6), can cross the blood–brain barrier and induce microglia activation.^42^ Moreover, pathological α-synuclein causes secretion of IL-6 by microglia and overexpressed IL-6 can induce toxic neuronal iron accumulation causing ultimately dopaminergic cell death.^43^ IL-6 levels are also elevated in CSF levels of PD patients and correlated positively with both anxiety and depression.^44^

Thus, new evidence strengthens the hypothesis that in addition to structural changes neuroinflammation can contribute to anxiety and depression in PD.

In line with this, non-steroidal anti-inflammatory drugs such as aspirin and celecoxib have been used in non-PD patients with depression.^45^

Cannabinoids, which are frequently used by PD patients for various non-motor symptoms, may also have anti-inflammatory properties.^46^ While some randomised placebo-controlled, double-blind studies showed that cannabinoids may improve anxiety^47,48^ others did not.^49^ The reason for this is probably differences in cannabinoid formulations, as well as different scales used to assess efficacy. Nevertheless, further studies are needed to evaluate the use of cannabinoids to alleviate anxiety in PD.^46^

## Evidence-based treatment of depression

Non-pharmacological approaches such as physical exercise, more specifically aerobic and resistance training should be considered in all PD patients and some may benefit from cognitive behaviour therapy. These non-pharmacological therapies may be even more useful in those PD patients with depression.^50,51^ Moreover, a systematic review showed that bright light therapy might also be efficacious in alleviating depression and improving sleep in PD.^52^ These approaches alone may be useful in those with mild symptoms.

The evidence for antidepressants in PD is overall good, although mixed results have been published (Table 2). Three randomised clinical trials including non-demented patients with PD and major depression demonstrated short-term effect of desipramine and citalopram within 4 weeks,^53^ of nortriptyline versus paroxetine or placebo within 8 weeks^54^ and of paroxetine and venlafaxine versus placebo over 12 weeks.^55^ When comparing routine treatment with transcranial magnetic stimulation, pramipexole or escitalopram all three therapies were superior to standard therapy alone. However, in this study, the best antidepressant effect as well as improvement in quality of life was achieved with escitalopram at a dose of up to 10 mg/day.^56^

**Table 2.** Evidence-based therapy of neuropsychiatric symptoms and insomnia in Parkinson’s disease.

Symptom	Medication/intervention	Dosage	Evidence level	References
Depression	Venlafaxine Escitalopram Paroxetine Nortriptyline Desipramine	37.5–225 mg 10 mg 10 mg 25–75 mg 75 mg	Recommended Possibly useful Possibly useful Possibly useful Possibly useful	55 56 55 53
Anxiety	Citalopram Desipramine Nortriptyline	20 mg 75 mg 50mg	Possibly useful Possibly useful Possibly useful	53 53 54
Apathy	Rivastigmine Piribedil	9.5 mg 50–300 mg	Possibly useful Possibly useful	74 78
ICDs	Reduction/cessation of DA Continuous dopaminergic therapy STN-DBS (in carefully selected patients)		Recommended Possibly useful Investigational	103
Hallucinations and psychosis	Clozapine Pimavanserin Quetiapine Donepezil/Galantamine/Rivastigmine (mild hallucinations plus dementia)	6.25–50 mg 34 mg 25–100 mg 5–10/16/12 mg	Recommended Recommended (US) Possibly useful Investigational	118 118 153
Insomnia	Melatonin Eszopiclone	3–5 mg 1–3 mg	Possibly useful Possibly useful	118 118
RBD	Melatonin Clonazepam	3–12 mg 0.5–2 mg	Recommended Recommended	118 118
MCI	Donepezil/rivastigmine	5–10/12 mg	Investigational/off label use	135, 136
Dementia	Rivastigmine Donepezil/Galantamine	3–12 mg 5–10/16 mg	Recommended Possibly useful	118

DA, Dopamine agonists; DBS, deep brain stimulation; ICDs, impulse control disorders; MCI, mild cognitive impairment; RBD, REM sleep behaviour disorder; STN, subthalamic nucleus.

In general selective serotonin reuptake inhibitors (SSRI), serotonin-norepinephrine reuptake inhibitors (SNRI) and tricyclic antidepressants have comparable effect sizes in PD. However, side effects of tricyclic antidepressants, particularly cognitive impairment, orthostatic hypotension as well as cardiac arrhythmia are clinically a limiting factor.

Thus, first-line therapy may consist of psychotherapy alone in mild cases or in combination with an SSRI or SNRI. Sometimes, especially in more severely depressed patients a combination of both drugs may be necessary. Among SSRIs, sertraline has been considered as the safest option.^21^ Prolonged QTc interval may limit the use of some antidepressant agents such as citalopram.^57^

There is insufficient evidence for other noradrenergic agents, such as reboxetine and atomoxetine.^22^ Duloxetine may be an exception as recently a systematic review reported effectiveness of duloxetine versus placebo on various depression scales. Moreover, duloxetine may also improve cognition in PD, although these findings need to be interpreted with caution because of the retrospective study design.^58^

Physicians should be aware of antidepressant induced movement disorders, such as bruxism, akathisia, myoclonus, dystonia, parkinsonism, restless legs syndrome, tremor and tics. These side effects were seen with several different antidepressants including venlafaxine, mirtazapine, citalopram, paroxetine, duloxetine, bupropion, clomipramine, escitalopram, fluoxetine and sertraline.^59^ In severe and drug refractory cases, electroconvulsive therapy may be considered, but only after careful specialist psychiatric evaluation.^21^

## Pragmatic therapy of anxiety

So far there is a lack of randomised controlled trials for anxiety in PD. Thus, optimising dopaminergic therapy by improving motor fluctuations, postural stability, freezing of gait or autonomic symptoms is particularly important in these patients.^60^

Similar to depression physical therapy and referral for cognitive behavioural therapy may be considered.^50,51^ Moreover, external stressors, such unfamiliar surroundings and social isolation should be avoided.^9^ One randomised controlled trial reported that 4 weeks of acupuncture may significantly improve anxiety in PD.^61^ However, the short study duration, the Asian population used, where the placebo effect of acupuncture may be present limit the interpretation of these results.

Several studies used anxiety as a secondary endpoint with conflicting results. One study showed that desipramine 75 mg/day and citalopram 20 mg/day were significantly better than placebo in alleviating anxiety at 30 days of follow-up.^53^ In contrast, another study failed to show a superior effect for citalopram and venlafaxine compared to placebo.^55^ Despite the lack of convincing data of SSRI’s, SNRI’s are often recommended (Table 2). In patients with depression and anxiety combined with insomnia, the antidepressants trazodone and the alpha 2 adrenoreceptor antagonist mirtazapine may be used.^60^ Low doses of benzodiazepines such as alprazolam, lorazepam or clonazepam may also be considered, but side-effects such as an increased risk of injuries and worsening of gait and balance may limit its use particularly in the older patients.^62,63^

## Apathy

Apathy is defined as a quantitative and persistent reduction in goal-directed activity in comparison to the patient’s previous level of functioning affecting behaviour, cognition, emotion and social interaction.^64^ It can be divided into four different subdomains: decrease in emotional resonance (reward deficiency syndrome), depression, decrease in cognitive interests (executive dysfunction) and absence of spontaneous activation of mental processes (auto-activation deficit).^65^ Clinically, these patients have a reduced initiative, emotional bluntness, a decreased interest in their surroundings and do not engage in social activities. Apathy is frequently found with an overall prevalence of about 40% for all PD stages.^66^ Moreover, it may affect up to a third of all de novo PD patients resulting in a significant impairment to patients’ daily life functioning. It has been more frequently seen in PD than in equally disabled patients with other chronic illnesses, suggesting that neuronal degeneration plays a pivotal role.^19^ In PD, apathy has been associated with cognitive impairment, but in contrast to previous studies, no link between apathy and disease progression was observed.^19^ Although apathy can present in isolation it frequently co-exists with other mood disorders, such as depression, anxiety or ICDs.^67^ Importantly, when present in isolation, apathy is distinct from depression, as apathetic but non-depressed patients do not report sadness, worthlessness or guilt.^67^

## Pathophysiology

Structural and anatomical imaging studies demonstrated dysfunction within the prefrontal cortex-striatal circuits in patients with apathy.^65^ Specifically, grey matter volume loss within the nucleus accumbens, the anterior cingulate was found in PD patients with compared to those without apathy. Moreover, a higher functional connectivity between the nucleus accumbens and dorsal anterior cingulate cortex was present in PD patients who later on developed apathy compared to those who did not.^68^ These findings suggests that dysfunction of functional connectivity may be a harbinger of apathy in PD^68^ and that dysfunction of structures important for reward processing may play a pivotal role in apathy.

## Experimental therapies for apathy

Treatment approaches include oxytocin, which is a neuropeptide that binds to oxytocin receptors in limbic and mediofrontal regions and may increase the salience of social cues. Moreover, oxytocin plays an important role in social interaction and controlling emotions. A recent study showed that low doses of intranasal oxytocin have a small but significant effect on apathy, albeit in patients with frontotemporal dementia.^69^ Given the overall few side effects and its potential neuroprotective effects,^70^ oxytocin may be an interesting drug to investigate for patients with PD and apathy.

One small open label study reported that the vortioxetine, a serotonin reuptake inhibitor and antagonist of 5-HT3, 5-HT7, 5-HTID; partial agonist of 5-HT1B may improve apathy after 3 months of therapy. In addition an improvement in depression, as well as fatigue and quality of life was observed. However, the open label study design limit interpretation of this study, and further trials are necessary to confirm these preliminary findings.^71^ One promising phase IIb randomised controlled trial demonstrated good safety and tolerability of pirepemat for apathy in PD patients with dementia. Pirepemat, a small molecule with possible effects on cortical neurotransmitters, such as norepinephrine, reduced apathy at 4 weeks.^72^ Further follow-up studies are currently under way.

## Evidence based therapy of apathy

There are no existing guidelines for the successful pharmacological or non-pharmacological treatment of apathy in PD (Table 2). This is mainly because of the heterogeneity of scales used to assess apathy and the short duration of the studies.^73^ Moreover, often apathy overlaps with depression and thus disentangling treatment effects may be difficult.

One double-blind placebo controlled study with 31 PD patients, who had no dementia or depression found that 6 months of treatment with rivastigmine at a dose of 9.5 mg/day could improve apathy compared to placebo.^74^ Likewise, albeit using different scales and in PD patients with dementia, apathy also improved following 20 weeks of treatment with rivastigmine. Interestingly, the effect of rivastigmine was greater in those patients with additional neuropsychiatric symptoms, particularly anxiety and delusions.^75^

Post-operative apathy is frequently observed following DBS of the STN. This post-DBS apathy seems to be independent of disease progression and presence of other neuropsychiatric symptoms.^76^ It has been hypothesised that a reduced mesolimbic dopamine levels due to a reduction of dopaminergic medication may be a contributing factor for developing apathy after DBS. In line with this, higher dopamine agonist doses have been shown to be associated with lower apathy scores in some^77^ but not all studies.^76^

Nevertheless, in patients with apathy following DBS, a trial of the dopamine D2, D3 receptor agonist piribedil may be useful. One randomised, double-blinded placebo controlled study found that apathy was significantly lower in the pribedil group compared to placebo after 12 weeks.^78^ Similarly, in open label trials apathy improved following treatment with other dopamine agonists, such as pramipexole as well as rotigotine.^79,80^ However, non-specific scales for apathy were used, and thus, these study offer low evidence.^65^ The role of antidepressants for apathy when without additional depression is controversial. One open labeled study showed a slight but non-significant improvement of apathy scores following paroxetine, duloxetine or citalopram with no difference between the SSRI and the SNRI group. However, a placebo arm was missing, which limits the interpretation of these findings.^81^

Non-pharmacological interventions such as cognitive behavioural therapy was successful in a small study improving apathy scores.^82^ The use of exercise therapies is conflicting with dancing and Nordic walking showing improvements in apathy scores,^83,84^ while circuit training, boxing and table tennis did not.^85–87^ It remains unclear why only some of these non-pharmacological interventions seem to be efficacious. Further and future technologically enhanced studies may be promising in the treatment of apathy.^88^

## Impulse control disorders

Behavioural addictions are common drug-induced side effects that are usually triggered by dopamine agonists and are collectively termed ICDs. ICDs are defined as a ‘failure to resist an impulse, temptation, or drive to perform an act that is harmful to the person or others’.^89^ In PD, compulsive sexual disorder, gambling disorder, compulsive shopping, and binge eating are the most common addictions. Other albeit less frequently seen addictions include the dopamine dysregulation syndrome (DDS), where patients take a much larger amount of levodopa against the physician’s advice and punding, which is the urge to perform senseless activities repeatedly (such as collecting or sorting objects).^8^ Although several risk factors for developing ICDs have been identified it is still unclear why only a subgroup of patients develop these addictions. Amongst non-pharmacological risk factors, using Genomewide Association Study, four novel genetic variants (*DAB1*, *PRKAG2*, *MEFV* and *PRKCE*) were identified.^90^ Other factors include a younger age, younger disease onset, being single, a novelty seeking personality trait, experiencing more non-motor symptoms as well as anxiety and depression.^8,91,92^

## Pathophysiology

The main risk factor for developing ICDs are dopamine agonists. It is likely that dopaminergic medication, especially dopamine agonists induce an ‘overdose’ of the ventral striatum in those with addictive behaviours. An immunohistochemistry study has shown that the nucleus accumbens had significantly less alpha-synuclein pathology in the nucleus accumbens compared to patients without addictions.^93^ This suggests that ventral striatum is better preserved in patients with ICDs. Moreover, dopamine agonist also reduce the striatal interaction to the prefrontal cortex resulting in an impaired cortical inhibition. Therefore, a combination of reduced inhibitory control and a prefrontal cortical dysfunction may be responsible for triggering ICDs in susceptible patients.^8^

## Experimental therapy

A promising target to improve impulsivity may be by modulating noradrenergic transmission with clonidine, an alpha-2 adrenergic agonist. Clonidine is approved for withdrawal symptoms and for the treatment of attention-deficit hyperactivity disorder in children.^94^ A multicentre phase IIB study in PD patients with various ICDs assessed the effect of clonidine on impulsivity. It was hypothesised that clonidine may improve inhibitory control. A non-significant greater and faster reduction of the total on the Questionnaire for Impulsive-Compulsive Disorders in Parkinson’s Disease-Rating Scale score was seen in the clonidine group compared to placebo at 8 weeks. Although the study was negative, the results of this study warrant a longer treatment duration and a larger sample size in a further phase III trial.^95^

Recently, a randomised, placebo-controlled, phase II trial (NCT03947216) assessing the effect of pimavanserin, a serotonin type 2A receptor (5HT 2A) inverse agonist, on ICDs has completed and results are expected within 2025.

Other dopaminergic formulations with a different receptor profile are also under investigation that may be useful in PD patients with ICDs. For example tavapadon, a novel dopamine agonist that targets dopamine D1/D5 receptors is now being tested in four clinical trials,^96^ may be useful in alleviating motor symptoms without triggering addictive behaviours.

Other mechanisms may warrant more exploration. Despite the recently published phase III randomised placebo-controlled trial which found no evidence that the glucagon-like peptide-1 (GLP-1) agonist exenatide may act disease-modifying,^97^ these drugs may be useful in improving addictive behaviours. For example, a reduction of alcohol intake and heavy drinking days compared to placebo^98^ as well as a reduction in substance abuse and binge eating was reported in the non-PD population.^99,100^ Recently, it has been hypothesised that GLP-1 agonists may also have effects on the reward-related behaviours and craving by reducing cortical activation in response to external cues.^101^ As such, GLP-1 agonists may be worth exploring as a potential therapy for addictive behaviours in PD.

## Evidence-based therapy for ICDs

Treatment of ICDs and DDS is often challenging due to low insight. Thus, prevention is key and patients as well as their family members should be aware of potential personality changes that may happen during treatment over time. Moreover, younger male patients, who have a personal or family history of addictive behaviours, or those who experience more motor symptoms such as dyskinesias as well as non-motor symptoms should be aware that they are at higher risk for developing ICDs. Screening and treatment of other additional neuropsychiatric symptoms and insomnia is particularly important in this cohort^8^ and may also play an important role in the long-term outcome. Improving of nighttime sleep as well as treating additional neuropsychiatric symptoms is especially important in these patients.^102^ Recently an expert committee has published a guideline for the management of ICDs and DDS in PD.^103^

If an ICD has been diagnosed treatment should depend on severity. In rare cases, a watch and wait approach or non-pharmacological approached such as cognitive behavioural therapy or limit access to money may be feasible. In the majority of cases, however, the addiction may have devastating financial and psychological consequences for the lives of patients and their families, if left untreated. Depending on the severity and type of addiction, dopamine agonist therapy should be reduced and eventually if necessary completely stopped.^8,103^ Around 20% of patients may develop dopamine agonist withdrawal symptoms, which may present with significant worsening of motor and non-motor symptoms. Particularly, neuropsychiatric symptoms and pain may limit withdrawal of dopamine agonists and hospital admission is often required. These symptoms may last between a few days or may be persistent and thus impair quality of life.^103^ Rotigotine is three times less likely to trigger ICDs compared to the oral dopamine agonists.^104^ Therefore, a transient low dose of rotigotine patch can be considered where withdrawal symptoms are intolerable. However, patients and their relatives must be aware of the risk of ongoing or remerging addictive behaviours. Thus, in patients with severe ICDs a reduction and cessation of dopamine agonist therapy is necessary to avoid the long-term devastating effects of these addictions. This is particularly import as insight is often low in these patients.^8^ Moreover, the link between dyskinesias and ICDs has been established in several studies. Thus, continuous anti-Parkinson drug therapies, either subcutaneously or via an intestinal gel infusion, should be considered in patients where reduction of dopaminergic therapy is met with great resistance.^8^ Several, albeit smaller, studies have shown that a continuous drug delivery, leading to more stable blood plasma levels, may not worsen or even improve ICDs in PD.^105–107^ In carefully selected patients DBS of the STN may be considered,^108^ although further high-quality studies are needed to confirm the use and safety of DBS in this patient cohort.^103^

Treatment of patients with DDS is particularly difficult as insight is usually very low in these patients. Moreover, there are no clear clinical guidelines. Hospital admission is often necessary in order to reduce dopaminergic dose, particularly fast acting levodopa or apomorphine pen injections.^8^ Fractionation of levodopa and treatment of additional anxiety and depression or the use of quetiapine or clozapine should be performed. However, these patients often do not tolerate lowering their dopaminergic medication due to worsening of motor fluctuations, ‘off’ dystonia or severe withdrawal symptoms. Thus, the prognosis of DDS is overall not good, with remission being achieved in only half of the patients. Those patients who have more motor fluctuations and having dyskinesias or being single having worse outcome.^103,109^ As mentioned above, a continuous drug delivery via a pump system or STN-DBS may be considered in these patients.

Management for punding behaviour in PD are not available. Usually, punding co-occurs with DDS but can also be present in isolation. In such patients, a reduction of bedtime levodopa and rescue levodopa doses, followed by a reduction of dopamine agonists should be aimed for^110^ (Table 2).

## Insomnia and REM sleep behavioural disorder

Sleep disturbances are frequently seen and mainly compromise insomnia, disorders of daytime somnolence, sleep-related breathing disorders, circadian disorders, restless legs syndrome and parasomnias. They affect a large proportions of patients with figures ranging from 64% to 90% and are more prevalent in PD patients with ICDs.^102,111,112^ Insomnia, with difficulties falling asleep, sleep maintenance and waking early as well as disturbances of the circadian rhythm are common and are often accompanied by neuropsychiatric symptoms such as depression, anxiety and cognitive decline.^111,112^ REM sleep behavioural disorder (RBD) is particularly relevant as it may precede PD for up to a decade and occurs in a quarter of de novo PD patients.^113^ The prevalence increases further with disease duration and may affect almost halve of patients during the course of the disease, with a female predominance.^112^ Clinically, can be lead to injuries RBD has been linked with a higher prevalence of neuropsychiatric symptoms such as cognitive impairment and hallucinations, which further reduces quality of life of these patients.^114^ Diagnosis of RBD requires a full night laboratory polysomnography, demonstrating REM sleep without atonia, defined as excessive phasic and/or tonic muscle activity in REM sleep.^115^

## Pathophysiology

Physiological sleep atonia is modulated by the amygdala and the coeruleus nucleus, which inhibit the cells of the spinal anterior horns, resulting in loss of skeletal muscle tone. It has been postulated that the reduction of signal intensity in the locus coeruleus is responsible for the development of RBD. Moreover, structural imaging in RBD patients revealed grey volume loss in several brain regions including the posterior cingulated gyrus and the hippocampus^116^ and a dopaminergic cell loss in. In line with this, a pathological hyperechogenicity of the substantia nigra has been reported in RBD.^116^ In PD, it was found that patients with additional RBD had a more profound and faster decline in white matter volume compared to PD controls, highlighting that additional RBD is a prognostic poorer sign.^117^

## Evidence-based therapy for insomnia and RBD

Non-pharmacological therapies such as cognitive behavioural therapy, exercise or bright light therapy should be considered. Moreover, drugs that may elicit sleep disturbances such as SSRI, SNRI or tricyclic antidepressants should be weaned off and dopaminergic therapy should be optimised to reduce ‘off’ periods or dyskinesias.

Melatonin (3–5 mg) and eszopiclone (1–3 mg) can improve insomnia compared to placebo are now both being considered as possibly useful. However, patients and their carers should be informed about the infrequent but serious risk of falls and fractures associated with eszopiclone.^118^ Trazodone (75–150 mg) is often used for insomnia but can have side effects such as drowsiness and drug-induced parkinsonism.^115^ Modafinil at a dose of 200–400 mg has been shown to improve daytime sleepiness, other therapies such as caffeine are still considered as investigational.^118^

Melatonin at higher doses (between 3 and 12 mg) is now the first-line therapy due to a better safety profile and fewer side effects such as falls and sedation than clonazepam (0.5–2 mg). However, both melatonin as well as clonazepam are effective in reducing RBD in PD (Table 2).

## Mild cognitive impairment and PD dementia

Mild cognitive impairment (MCI) represents a cognitive profile that is not normal for age and has been recognised as a prodromal state for Parkinson’s disease dementia (PDD). MCI occurs in approximately 10%–20% of people with PD at the time of diagnosis and is often underdiagnosed. Clinically, cognitive dysfunction in PD is heterogeneous with non-amnestic impairment, such as executive and visuospatial as well attention deficits being more common than decline in memory. Additional non-motor symptoms, such as apathy, depression, fatigue, hallucinations or changes in the sleep-wake cycle may be present.^119^ Moreover, PD patients with MCI have a high risk of developing dementia. Yet, there are also exceptions, where cognition may remain stable for several years and in some cases, may even improve to normal.^120^ Risk factors for conversion to dementia include, older age, severity of motor symptoms, speech impairment, early autonomic symptoms and poorer response to dopaminergic therapy as well as confusion, hallucination on dopaminergic medication and psychosis.^120,121^

In line with this, the presence but not necessarily the severity of mood disorders such as depression, psychosis and apathy is often associated with cognitive impairment and Parkinson dementia.^19^ Specifically, mood disorders correlated with lower scores on verbal memory tasks and executive function, while aggression and disinhibition was associated with visuospatial dysfunction and naming deficits.

The cross-sectional proportion PD patients with frank dementia is 24%–31%. Heterogeneous results have been reported on the cumulative prevalence of PDD and vary between 30% and 50%.^122,123^ Reasons for this variation may include demographic and cultural differences as well as different inclusion criteria and assessment tools used.^122^ However, the risk of developing dementia increases with disease duration and the vast majority of patients (around 80%) will have dementia within 20 years after diagnosis.^123^

## Pathophysiology

A variety of different hypothesis on the mechanisms for cognitive changes in PD have been proposed. Most importantly, co-existing Alzheimer pathology with extracellular β-amyloid in cortical and subcortical regions and tau pathology in hippocampal and neocortical regions is common in those with cognitive impairment.^123^ These patients have also a higher risk of amyloid angiopathy and a more rapid decline than PDD patients without Alzheimer’s disease pathology.^124^ Furthermore, alpha-synuclein deposition in the limbic and neocortical regions (frontal and temporal) are also common in PD patients with cognitive decline. Functional changes within the dopaminergic pathways and a more severe dopaminergic cell loss may also play a prominent role cognitive dysfunction in PD. For example, a more widespread degeneration of dopaminergic terminals in the striatum has been found in PD patients with MCI compared to PD patients without cognitive deficits. Those with PDD have additional loss within the frontal, parietal and temporal regions.^123^ In line with this, noradrenergic cell loss has been also associated with cognitive decline in PD. Locus coeruleus imaging has been therefore proposed as a promising biomarker for early detection for cognitive decline in PD.^125^

Higher neurofilament light chain protein in CSF, a lower hippocampal volume and more non-motor symptoms such as depression and RBD have been also identified as risk factors for faster cognitive decline.^126^ Moreover, genetic risk factors include variants in the *GBA* gene, multiplications in the α-synuclein gene (*SNCA*), H1 haplotype of the *MAPT* gene and carrying the *APOE 4* allele.^120^ In line with this, risk factors for conversion to PD dementia also include the *APOE 4* allele, mutations in the *GBA* gene and reduced levels of amyloid beta 42 in the CSF.^127^

## Evidence-based therapy for MCI and PDD

A thorough history to outline the onset and the rate of cognitive decline is important as well as identifying precipitating factors. As mentioned above, secondary causes should be treated and drugs that may decrease cognitive functions (e.g. sedatives, anticholinergic agents, opioids) should be weaned off. Other strategies, such as improvement of nighttime sleep and avoidance of low nighttime lighting to reduce visual misperception may be useful in reducing confusion.

Despite conflicting results cognitive training may be additionally efficacious in slowing down cognitive decline^128,129^ or even improving some aspects of cognition^130,131^ although these effects seem not be long-lasting.^132^ Nevertheless, a trial of cognitive training may increase quality of life and depression.^130,131^ Physical exercise,^133^ particularly dual-task performance with motor and cognitive training should also be recommended. More specifically, an increase in gait speed, cadence as well as stride length and a decrease in dual-task cost on gait speed was observed in a recent meta-analysis.^134^

No approved pharmacological management is available for PD patients with MCI. Several studies, albeit with a small sample size or short trial duration, have shown that acetylcholinesterase inhibitors, such as donepezil at a dose of 10 mg and rivastigmine at 12 mg may have some positive effects on cognitive function without worsening parkinsonism.^135,136^ In line with this, PD patients treated with donepezil at a dose of 5 mg scored higher on the Mini-Mental State Examination (MMSE) and on an auditory memory task over a period of almost 2 years compared to those treated with placebo. However, donepezil had no prophylactic effect on development of psychosis.^137^ Furthermore, early use versus a delayed start of donepezil led to a significantly less steep decline in the former group, particularly in those who were apolipoprotein ε4 carriers.^138^ Donepezil may also have positive effects on gait. For example, those treated with donepezil 10 mg treated for 6 weeks fell approximately half as often as those in the placebo arm,^139^ and similar results were reported with rivastigmine, probably due to improved attention.^140^ Thus, additionally to non-pharmacological strategies and despite limiting evidence a trial with donepezil at a dose of 10 mg or rivastigmine may be considered in PD patients with MCI.

The only FDA- and EMA-approved therapy for PDD is rivastigmine, based on a randomised controlled trial^141^ (Table 2). Despite both capsules and patches can improve cognition and behaviour oral formulations may have greater effects.^123^ The evidence for donepezil is conflicting,^142^ but discontinuation of acetylcholinesterase inhibitors in PDD may worsen clinical outcomes.^57^ There is insufficient evidence for memantine with conflicting results of various randomised controlled trials.^57^ Moreover, there is no clear evidence that neuromodulator treatments are useful for PD patients with MCI or dementia.^57^

## Hallucinations and psychosis

Hallucinations are common in PD, with visual hallucinations (VH) being significantly more common than other auditory, tactile or olfactory hallucinations. In fact, VH can affect up to 75% of patients over the disease course.^143^ They usually start with visual misperceptions to well-formed complex hallucinations where insight can be present but can evolve into a more pervasive psychosis with delusions.^144^ Thus, VH can start benign, but they can become distressing over the course. Typically, they are very detailed and involve people or animals and occur in familiar surroundings. Sometimes these hallucinations may involve the illusion of objects passing across the peripheries of vision or the sense of a presence.^143^ Not surprisingly, VH have been associated with cognitive impairment and with a more aggressive PD phenotype.^145^ More specifically, impairment in executive functions, attention, episodic memory as well as lower scores on verbal memory were reported in those with VH.^146,147^ Thus, in these patients a careful assessment for cognitive decline and dementia should be performed.

## Pathophysiology

Structural MRI showed that dysfunction of multiple brain areas rather than a focal lesion is responsible for VH.^148^ Heterogeneous results in cortical thickness with reduction in grey matter volume in the posterior and the parietal regions, more specifically in the cuneus, precuneus and middle occipital gyrus were found.^146,148^ More importantly, changes in functional connectivity with a decreased bottom-up and increased top down connectivity have been described. Particularly connectivity from the lateral geniculate nucleus to the prefrontal cortex and dysfunction of the connectivity from prefrontal cortex to primary visual cortex have been described in PD patients with VH compared to those without.^149^ While minor hallucinations have been linked to grey matter loss and hypometabolism in the posterior cortex, structured hallucinations and delusions are associated with additional atrophy of the hippocampus as well as the upper brainstem and the thalamus.^145,150^

Neurotransmitter dysfunction, mainly degeneration within the central cholinergic system may play an important role in VH. More specifically, degeneration of the pedunculopontine nucleus and the nucleus basalis of Meynert contain the largest amount of cholinergic neurons. With its wide connections to several brain areas, such as the frontal cortex, cingulate and amygdala, the posterior cortex and the thalamus sensory processing may be disturbed. Initially correction by engagement of attentional control networks may suppress thalamic sensory dysfunction. However, as the disease progresses, dysfunction of the nucleus basalis of Meynert with its projections to the frontal and parietal cortex may limit the ability to engage these control networks.^144^

Although dopaminergic medication can worsen VHs in PD, they may also occur in drug naïve patients.^151^ Thus, it is unlikely that dopaminergic medication is the main contributing factor for trigger VH. In contrast to VH, psychosis in PD rarely occurs in drug-naïve patients but is common as the disease progresses.

Moreover, psychosis is associated with poor prognosis such as greater physical disability, cognitive and affective dysfunction, caregiver distress, nursing home placement and mortality.

## Experimental therapy for hallucinations and psychosis

Novel therapeutic approaches, that are recently approved for the treatment of schizophrenia may also be effective in reducing hallucinations in PD. Ulotaront is a trace amine-associated receptor 1 agonist with 5-HT_1A_ agonist activity, without blocking dopamine D2 receptors. In an exploratory phase II pilot trial the ulotaront-treated PD group, especially those with cognitive impairment, showed a non-significant trend in reduced hallucinations compared to placebo at week 6. Interestingly, however remission rate was 25% in the treatment compared to 0% in the placebo arm. Ulotaront may therefore be a viable option for PD patients with psychosis without worsening motor symptoms.^152^

## Evidence-based therapy for hallucinations and psychosis

Psychotic symptoms do not always need treatment, particularly if they are not distressing and the patient has full insight.^153^ However, if treatment is required these patients should be ideally treated in their familiar surroundings. Screening for secondary causes, such as infections or dehydration should be performed. If secondary causes can be excluded, a thorough review of the current medication is necessary. Often accidental intake of extra dopaminergic doses can trigger psychotic symptoms.^153^ Screening for cognitive impairment is necessary and a trial of cholinesterase inhibitors in those with mild hallucinations and dementia should be considered.^153^

Moreover, drugs which can induce hallucinations or psychosis, such as anticholinergic agents, tricyclic antidepressants, benzodiazepines or opioids should be withdrawn. Anti-Parkinson medication such as amantadine, dopamine agonists, monoamine-oxidase inhibitors and catechol-O-methyltransferase should be weaned off. Finally, if necessary, a reduction of levodopa may be necessary.^145,153^ However, a reduction of dopaminergic medication may lead to intolerable worsening of motor symptoms or no sufficient improvement of symptoms. In these cases a trial of cholinesterase inhibitors or antipsychotic medication, such as quetiapine, clozapine and pimavanserin should be considered (Table 2).

Cholinesterase inhibitors, such as donepezil, rivastigmine and galantamine, have a small effect on psychotic symptoms in PD. Despite the conflicting studies,^153^ a recent meta-analysis including five trials in PD showed small but significant effect sizes in reducing hallucinations and psychosis in PD.^154^ In clinical practice, PD patients with cognitive impairment and additional psychosis may benefit most from treatment with a cholinesterase inhibitor.^153^

Quetiapine is often used in patients with delusions or minor hallucinations based on several retrospective reports.^153^ However, none of the randomised controlled trials have shown efficacy of quetiapine in improving hallucinations or psychosis in PD.^155–157^ Thus, the Movement Disorders Society Evidenced-Based Medicine Committee rated that there is insufficient evidence for quetiapine for the treatment of psychosis in PD but in clinical practice quetiapine is ‘possibly useful’.^118^ Side effects may include sedation or orthostatic hypotension and therefore if possible a low dose (e.g. 25 or 50 mg extended release) should be started.

Pimavanserin has been approved by the FDA in 2016 for the treatment of psychosis in PD but is only available in theUnited States. Although the exact mode of action is unclear it is believed the 5-HT2A receptor plays a prominent role in the development psychosis in PD.^158^ There are no head-to-head trials comparing pimavanserin to clozapine or quetiapine, but it seems that pimavanserin takes longer (around 4–6 weeks) until it shows an effect.^153^ Moreover, and in contrast to quetiapine and clozapine, little is known about the long-term safety.

Clozapine has been studied in randomised, double-blinded placebo-controlled trials as well as in other smaller chart reviews with doses in PD much lower than used in patients with schizophrenia.^153,159–161^ It has a high affinity to dopamine D1 and much lesser to dopamine D2 receptors. Moreover, it has an affinity for serotonergic 5-HT2A and 5-HT2C, which may explain the very low does (6.25–37 mg) used in PD.^145^ It is, however, important to perform regular blood cell monitoring, as clozapine has a very low risk of severe neutropenia and agranulocytosis and a low risk of mild and moderate neutropenia.^162^ Nevertheless, clozapine is the most efficacious drug for the treatment of hallucinations and psychosis in PD and is likely underused in most countries.^160^

## Conclusion

Neuropsychiatric symptoms remain challenging and need to be considered as a major part of the PD. Early detection and screening is of importance to address the multifaceted and complex symptoms that can significantly impair quality of life of patients and their carers. A multidisciplinary approach targeting the individual needs of patients and their family members is necessary for the successful treatment of neuropsychiatric symptoms in PD.

## Declarations

**Ethics approval and consent to participate:** Not applicable.

**Consent for publication:** Not applicable.

**Availability of data and materials:** Not applicable.
